# Whole-Genome Sequencing and *De Novo* Assembly of Malassezia pachydermatis Isolated from the Ear Canal of a Dog with Otitis

**DOI:** 10.1128/MRA.00205-21

**Published:** 2021-05-27

**Authors:** S. D’Andreano, J. Viñes, O. Francino

**Affiliations:** a SVGM, Molecular Genetics Veterinary Service, Universitat Autònoma de Barcelona, Bellaterra, Barcelona, Spain; b Vetgenomics, Edifici EUREKA, PRUAB, Bellaterra, Barcelona, Spain; University of California, Riverside

## Abstract

We have *de novo* assembled the genome sequence of Malassezia pachydermatis isolated from a canine otitis sample with Nanopore-only long reads. With 99× coverage and 8.23 Mbp, the genome sequence was assembled in 10 contigs, with 6 of them corresponding to chromosomes, improving the scaffolding of previous genome assemblies for the species.

## ANNOUNCEMENT

*Malassezia* (Eukaryota; Fungi; Basidiomycetes) is a lipid-dependent yeast of the skin mycobiome in humans and other mammals (1–3). The genus includes 18 closely related species described to date (4), and M. globosa, *M. restricta*, and *M. sympodialis* are the most common human skin residents, whereas *M. pachydermatis* is the most common species related to skin disorders in veterinary medicine (3, 5).

As of February 2021, there are only two assembled genome sequences for *M. pachydermatis*, under NCBI accession no. GCF_001278385.1 (6) and GCA_001264975.1 (7), which are both from the collection strain CBS 1879. There is a need to gather genomic information from *Malassezia* isolates associated with skin disorders important in animal and human health. We have assembled a high-quality *M. pachydermatis* genome sequence isolated from a dog with otitis using a *de novo* assembly and polishing strategy with Nanopore-only long reads.

Leti Animal Health kindly provided an isolate identified as *M. pachydermatis* after microbiological culture of a swab sample collected from a dog’s ear canal with otitis in Barcelona, Spain. The culture was grown at 28°C in dermatophyte test medium (DTM) agar and Saboraud-chloramphenicol agar. DNA was extracted with a Biomics DNA miniprep kit (Zymo Research, USA). DNA quality and quantity were determined using a Nanodrop 2000 spectrophotometer and Qubit double-stranded DNA (dsDNA) broad-range (BR) assay kit (Fisher Scientific SL, Spain). Before whole-genome sequencing, the isolate was confirmed as Malassezia pachydermatis by long-amplicon Nanopore sequencing of the fungal ribosomal operon and taxonomy assignment at the species level with the cloud-based WIMP application from the EPI2ME platform (Oxford Nanopore Technologies [ONT] Ltd., UK), as previously reported (8). The sequencing library was prepared with the 1D native barcoding genomic DNA kits (EXP-NBD103 and SQK-LSK109; ONT) and sequenced in a MinION FLO-MIN106 v9.4.1 flow cell in a MinION Mk1B instrument for 21 h, using the MinKNOW v18.12.9 software.

The fast5 files were base called with Guppy v2.3.7 (ONT), with the flip-flop algorithm for improved accuracy. Reads with a quality score lower than 7 were discarded. The fastq files were demultiplexed and trimmed with Deepbinner (9). We further *de novo* assembled the genome sequence with Flye 2.6 (10) and corrected the contigs with racon (https://github.com/lbcb-sci/racon) and Medaka (https://github.com/nanoporetech/medaka). We assessed the genome completeness with Benchmarking Universal Single-Copy Orthologs (BUSCO) v4.0.1 (https://gitlab.com/ezlab/busco) (11) in mode genome with basidiomycota_odb10 (20 November 2019; no. of species, 133; no. of BUSCOs, 1,764). We used Bandage (12) for contig visualization and comparison with the CBS 1879 *M. pachydermatis* reference genome (GCF_001278385.1) (6).

**FIG 1:**
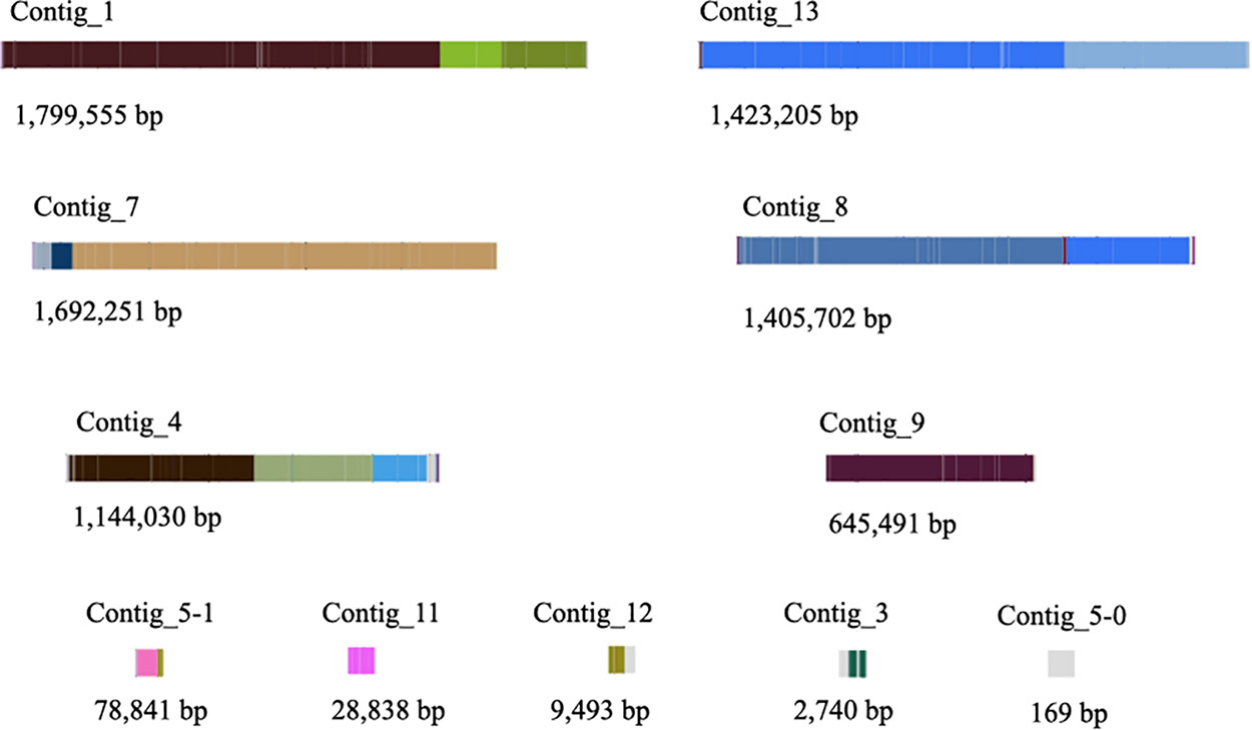
Bandage visualization of the genome assembly for *Malassezia pachydermatis* (GenBank accession no. GCA_014493585.1). Contig_1, Contig_13, Contig_7, Contig_8, Contig_4, and Contig_9 correspond to the six chromosomes of *M. pachydermatis*. Contigs are colored relative to the reference assembly for the CBS 1879 strain (GCF_001278385.1) (6) and indicate the merge of contigs that are separate from the CBS reference genome assembly with short reads.

Nanopore sequencing generated 265,298 reads with an *N*_50_ value of 5,648 bp and allowed successful *de novo* assembly and polishing of the genome sequence of *M. pachydermatis* (Table 1). Coverage was 99×, with a GC content of 54.54%. The genome was 8.23 Mbp in 10 contigs (longest contig, 1.799 Mbp; *N*_50_, 1.423 Mbp), with the longest ones corresponding to the 6 expected chromosomes (13) (Fig. 1). The average nucleotide identity (ANI) (14) was 96.3% with the *M. pachydermatis* reference genome (GCF_001278385.1), confirming the isolate as the same species (15, 16). The 76.6% BUSCO completeness (C,76.6% [S,76.5%; D,0.1%]; F,3.8%; M,19.6%; n,1,764) is similar to the *M. pachydermatis* reference genome with short reads (6, 7) and with the same duplication of the glutathione reductase gene as the reference genome.

**TABLE 1:** *De novo* assembly of the *Malassezia pachydermatis* genome sequence isolated from a canine otitis sample^*a*^

Sequence name	Sequence role	Assigned molecule	Assigned molecule location type	GenBank accession no.	Sequence length (bp)
Contig_1	Assembled molecule	1	Chromosome	CM025284.1	1,799,555
Contig_7	Assembled molecule	2	Chromosome	CM025285.1	1,692,251
Contig_13	Assembled molecule	3	Chromosome	CM025286.1	1,423,205
Contig_8	Assembled molecule	4	Chromosome	CM025287.1	1,405,702
Contig_4	Assembled molecule	5	Chromosome	CM025288.1	1,144,030
Contig_9	Assembled molecule	6	Chromosome	CM025289.1	645,491
Contig_11	Unplaced scaffold	NA	NA	JABNMX010000001.1	28,388
Contig_12	Unplaced scaffold	NA	NA	JABNMX010000002.1	9,493
Contig_3	Unplaced scaffold	NA	NA	JABNMX010000005.1	2,740
Contig_5	Unplaced scaffold	NA	NA	JABNMX010000007.1	74,481

a BioProject PRJNA631787, assembly GCA_014493585.1, accession no. JABNMX01, and isolate M13-UAB2019. NA, not applicable.

### Data availability.

The standardized strain descriptions and accession numbers are presented in Table 1; the genome assemblies and genomic data are publicly available in DDBJ/ENA/GenBank under BioProject no. PRJNA631787 with the assembly accession no. GCA_014493585.1 and genome accession no. JABNMX01. The version described in this paper is the first version. The raw data are available from the Sequence Read Archive (SRA) under the accession no. SRR13764144. Raw data (fast5) are available at Zenodo (https://zenodo.org/record/3874381#.YJQ1rLVKi70).
